# Body mass index, sitting time, and risk of Parkinson disease

**DOI:** 10.1212/WNL.0000000000005328

**Published:** 2018-04-17

**Authors:** Elin Roos, Alessandra Grotta, Fei Yang, Rino Bellocco, Weimin Ye, Hans-Olov Adami, Karin Wirdefeldt, Ylva Trolle Lagerros

**Affiliations:** From the Department of Medicine (E.R., Y.T.L.), Unit of Clinical Epidemiology, Karolinska Institutet, T2, Stockholm; Department of Medical Epidemiology and Biostatistics (A.G., F.Y., R.B., W.Y., H.-O.A., K.W.), and Department of Clinical Neuroscience (K.W.), Karolinska Institutet, Stockholm; Centre for Health Equity Studies (A.G.), Stockholm University/Karolinska Institutet, Stockholm, Sweden; Department of Statistics and Quantitative Methods (R.B.), University of Milano-Bicocca, Milan, Italy; Clinical Effectiveness Research Group (H.-O.A.), Institute of Health and Society, University of Oslo, Norway; and Department of Medicine (Y.T.L.), Clinic of Endocrinology, Metabolism and Diabetes, Karolinska University Hospital, Stockholm, Sweden.

## Abstract

**Objective:**

Causes of Parkinson disease are largely unknown, but recent evidence suggests associations with physical activity and anthropometric measures.

**Methods:**

We prospectively analyzed a cohort of 41,638 Swedish men and women by detailed assessment of lifestyle factors at baseline in 1997. Complete follow-up until 2010 was achieved through linkage to population-based registers. We used multivariable Cox proportional hazards models to estimate hazard ratios with 95% confidence intervals (CIs).

**Results:**

We identified 286 incident cases of Parkinson disease during follow-up. Multivariable adjusted hazard ratios were 1.06 (95% CI 0.76–1.47) for sitting time ≥6 vs <6 hours per day; and 1.13 (95% CI 0.60–2.12) for body mass index ≥30 vs <25 kg/m^2^. Results did not differ by sex.

**Conclusions:**

No association between prolonged sitting time per day or obesity and risk of Parkinson disease was found.

The etiology of Parkinson disease (PD) is unknown. Environmental^1^ and lifestyle factors including body mass index (BMI) are discussed in the context of PD causation.^2^ By decreasing dopamine receptor availability, obesity may increase the risk of PD.^3^

We recently reported that higher household and commute physical activity lowers the risk of PD.^4^ Regardless of time spent on physical activity, sedentary behavior characterized by sitting extended periods of time has been associated with increased general morbidity and mortality.^5^ Thus, sedentary behavior may affect PD pathogenesis through mechanisms other than physical activity. No previous study has, however, examined sitting time and the risk of developing PD. We therefore studied BMI and sitting time in a large cohort of Swedish men and women with a 13-year follow-up and detailed adjustment for physical activity and other potential confounders.

## Methods

### Study population

The Swedish National March Cohort comprises 43,863 Swedish participants who, at a national fund-raising event in September 1997, completed a comprehensive questionnaire with extensive assessment of lifestyle factors.^6^ Exposure data were collected for smoking, alcohol intake, height and weight, as well as education level. We also assessed physical activity and sedentary behavior using a questionnaire specially developed and validated for this study.^7^

All participants provided their individually unique national registration number, enabling identification of health status through linkage of medical records to Swedish population-based registers. Participants who lacked a valid registration number (n = 11), emigrated, died, had a PD diagnosis before start of follow-up (n = 482), or who were younger than 18 years (n = 1,732) were excluded. In total, 41,638 participants were finally included in the study.

Participants were followed from October 1, 1997, until a primary or secondary diagnosis of PD, emigration, death, or until end of follow-up on December 31, 2010, whichever occurred first. Index date of diagnosis was defined as first-ever outpatient contact or hospital discharge with a diagnosis of PD from the Swedish Patient Register. The Swedish revisions of the ICD codes for PD were used: 350 (ICD-7, 1964–1968), 342 (ICD-8, 1969–1986), 332A (ICD-9, 1987–1996), and G20 (ICD-10, 1997–2010).

### Statistical methods

Height and weight were self-reported in the questionnaire and BMI was calculated by dividing reported weight (kg) by the squared height (m^2^). BMI was categorized according to the standard classification of the World Health Organization into underweight or normal weight (<25 kg/m^2^), overweight (≥25 to <30 kg/m^2^), and obesity (≥30 kg/m^2^). Because few individuals were underweight (n = 579), we categorized both underweight and normal weight as “normal weight.” Sitting time was characterized by bathing, listening to music, watching television, office work, knitting, and sewing and categorized into either less or more than 6 hours of sitting per day.

We first assessed the baseline characteristics of the study participants for the entire cohort and stratified by BMI and sitting time. We computed age-standardized incidence rates for PD in each category of BMI and sitting time. Direct standardization was implemented using the 5-year age categories distribution of follow-up person-years of the entire cohort.

A Cox proportional hazards regression model was fitted to estimate hazard ratios and corresponding 95% confidence intervals (CIs) of PD at various levels of BMI and sitting time, using age as the underlying time scale. We initially adjusted for sex. Then we further adjusted for the following potential confounders: smoking status (never, former, current), alcohol consumption (<73.3, ≥73.3 to <314.8, or ≥314.8 g/mo), coffee intake (never, low [1–2 cups/d], medium [3–4 cups/d], or high [≥5 cups/d]), and educational level (≤13 or >13 years), and waist circumference categorized into standardized categories for women (<80, ≥80 to <88, or ≥88 cm) and men (<94, ≥94 to <102, or ≥102 cm), according to the International Diabetes Federation consensus and the World Health Organization. Participants reported how many hours per week they spent on household activities, such as working in the garden, cleaning the house or commuting, such as walking or biking to work. We were therefore able to further adjust for household and commuting physical activity (≤2, 3–4, 5–6, >6 h/wk) as our finding in a previous study showed an inverse association with this type of physical activity and risk of PD.^4^

We assessed a linear trend across categories of BMI and sitting time by including the median values of each category of BMI and sitting time in the model as a continuous variable. We also repeated the analyses stratified by sex, smoking status (never, ever), and age at baseline (<65, ≥65 years). We used the deviance test based on scaled Schoenfeld residuals to assess the proportional hazards assumption and ran stratified Cox regression models when the assumption was not satisfied. Finally, we performed 3 sensitivity analyses. First, we excluded the first 2, then 3, and then 5 years of follow-up to avoid reverse causation bias. In the second one, we also included PD cases identified through the Cause of Death Register. In a third sensitivity analysis, we used multiple imputation models based on chained equations^8^ under the assumption of data missing at random to assess the robustness of the observed findings. We imputed missing values only for the confounders.

The proportion of missing data on the exposure variables was 4.6% for BMI, and 2.0% for sitting time. Percentages of missing values on confounders were all below 2%, except for smoking and waist circumference, which were missing for 8.4% and 24.6% of the study participants, respectively.

The *p* values <0.05 were considered statistically significant. All statistical analyses were performed with Stata version 14 (StataCorp LP, College Station, TX).

### Standard protocol approvals, registrations, and patient consents

The research ethics board in Stockholm approved the study and all subjects gave informed consent.

## Results

Participants' characteristics are presented in table 1. Participants who were obese had a lower educational level compared to both normal and overweight participants. Those who sat 6 or more hours per day were more likely to be ever smokers, high consumers of alcohol, and to have a higher educational level compared to participants who sat less than 6 hours a day.

**Table 1:**
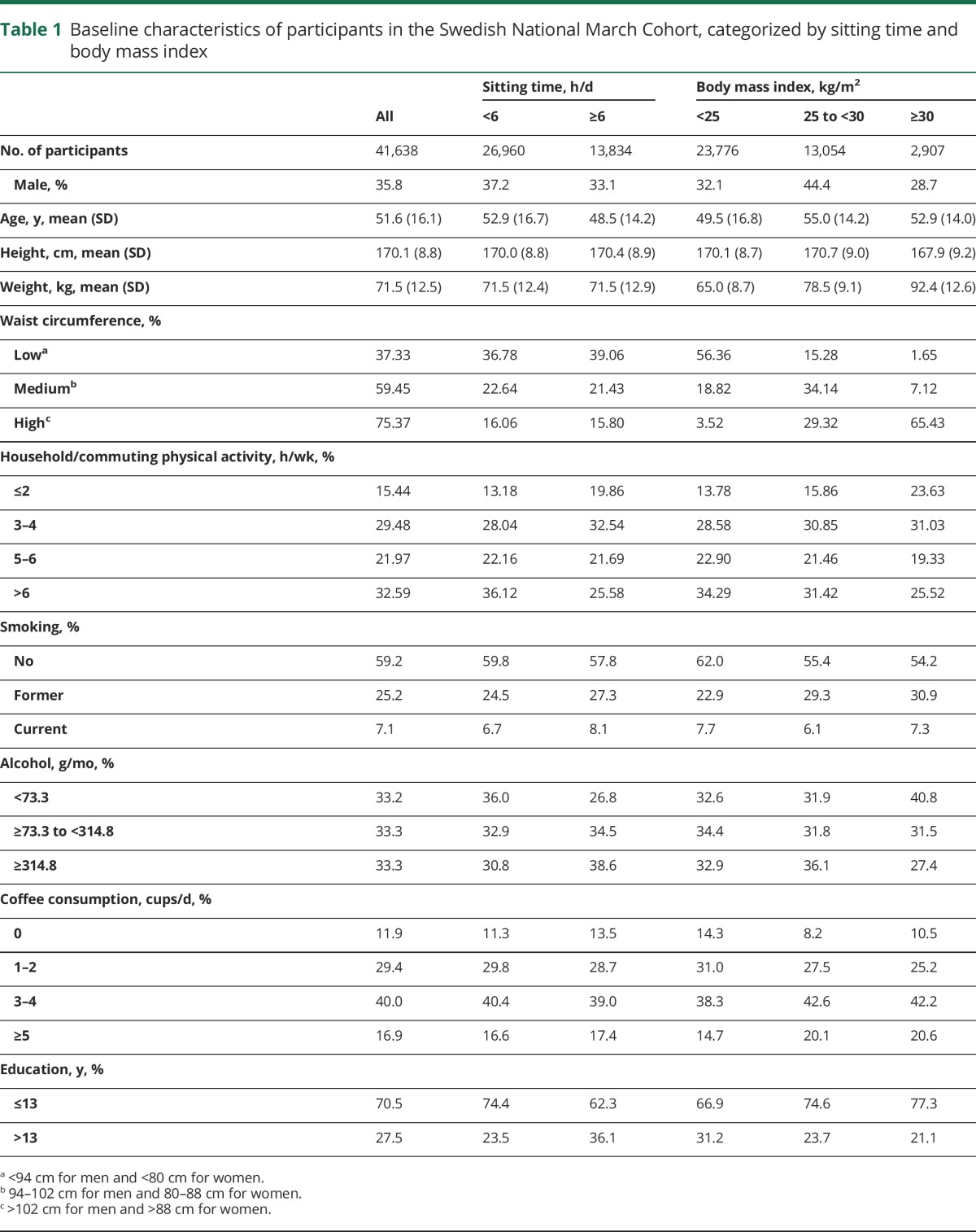
Baseline characteristics of participants in the Swedish National March Cohort, categorized by sitting time and body mass index

We identified 286 incident cases of PD during 159 months of follow-up. We found no significant association between baseline BMI, sitting time, and risk of PD, or any evidence of trend (table 2). Age- and sex-adjusted analyses provided results similar to our multivariable adjusted results presented in table 2 (results not shown). In these analyses, sitting time of 6 or more hours per day compared with less than 6 hours per day increased risk of PD by 6% (95% CI −24% to 47%), and BMI of 30 or higher compared with BMI less than 25 increased risk of PD by 13% (95% CI −40% to 112%). When we stratified the analyses for sex, smoking status (ever-never), and age at baseline (cutoff 65 years), the results were similar in men and women. The results from the sensitivity analyses did not differ significantly (results not shown).

**Table 2:**
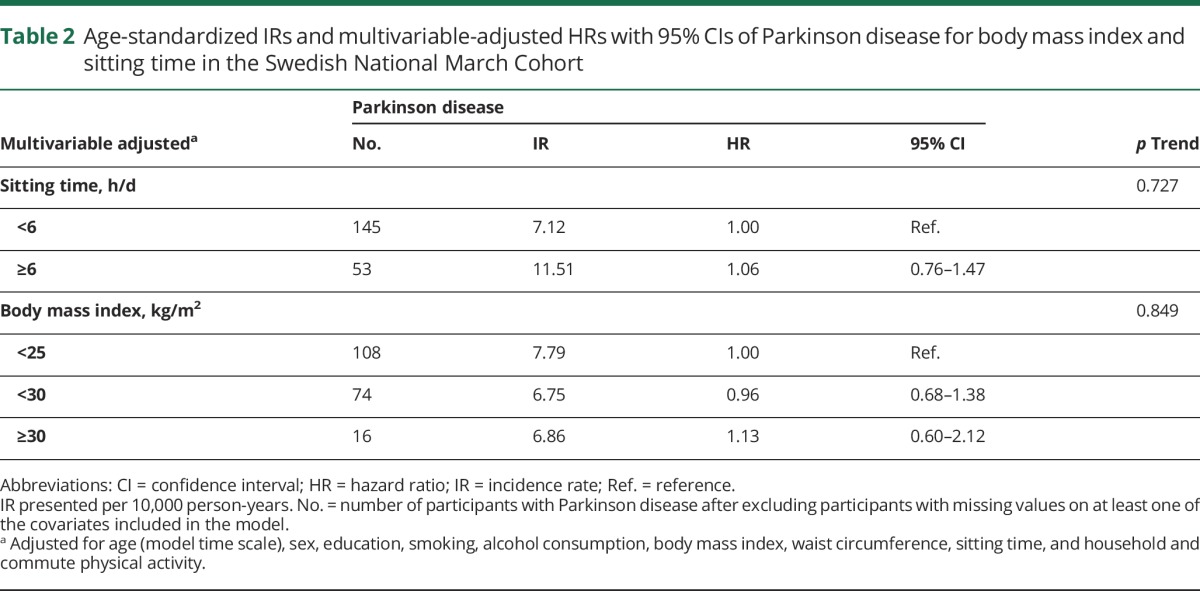
Age-standardized IRs and multivariable-adjusted HRs with 95% CIs of Parkinson disease for body mass index and sitting time in the Swedish National March Cohort

## Discussion

In this prospective cohort study, we found no association between sitting time or BMI and risk of PD. Associations between sedentary behavior and vascular diseases, depression, and impaired cognitive function have been described.^5^ Studies examining the relationship between sitting time and PD risk are rare. Palacios et al.^9^ reported no relationship between BMI and PD. Our findings are in line with both Palacios et al. and a recent quantitative review showing no evidence that high BMI increases the risk of PD.^10^

Strengths of our study include its prospective design, complete long-term follow-up, inclusion of both men and women, and extensive adjustment for potential confounders. The limited statistical power of the study undermines its ability to establish weak associations. Like many other epidemiologic studies, we relied on self-reported information for body measures potentially inducing misclassification. Such misclassification would be nondifferential and lead to an attenuation of the association given our prospective study design. Also, validation studies of self-reported weight and height have shown that BMI values calculated from self-reports have a high sensitivity in classifying someone as overweight or obese.^11,12^

Nevertheless, anthropometric measures as well as sitting time might have changed during follow-up. If such changes are related to the outcome, this could lead to misclassification and an over- or underestimation of any true association. Still, if PD development begins many years before symptom onset, this places the exposure window of interest closer to the baseline exposure assessment. Repeated exposure assessments closer to the end of follow-up might increase the risk of reverse causation.

The diagnosis of PD was obtained from the Swedish Inpatient Register and although the accuracy is good,^13^ misclassification between PD and other parkinsonian disorders might have occurred, and information on the relative severity at time of diagnosis and symptom onset is not available in the Inpatient Register.

Our findings indicate that sitting time and BMI are not associated with PD risk. Future studies should focus on environmental factors other than obesity and sedentary time in efforts to disentangle the complex causation of PD.

## Glossary

BMI: body mass index
CI: confidence interval
ICD: *International Classification of Diseases*
PD: Parkinson disease
